# Global organization of a binding site network gives insight into evolution and structure-function relationships of proteins

**DOI:** 10.1038/s41598-017-10412-z

**Published:** 2017-09-14

**Authors:** Juyong Lee, Janez Konc, Dušanka Janežič, Bernard R. Brooks

**Affiliations:** 1 Department of Chemistry, Kangwon National University, 1 Kangwondaehak-gil, Chuncheon, 24341 Republic of Korea; 2 Laboratory of Computational Biology, National Heart, Lung, and Blood Institute, National Institutes of Health, Bethesda, Maryland 20892 United States; 3 Faculty of Mathematics, Natural Sciences and Information Technologies, University of Primorska, Glagoljaška 8, SI-6000 Koper, Slovenia; 4 National Institute of Chemistry, Hajdrihova 19, SI-1000 Ljubljana, Slovenia

## Abstract

The global organization of protein binding sites is analyzed by constructing a weighted network of binding sites based on their structural similarities and detecting communities of structurally similar binding sites based on the minimum description length principle. The analysis reveals that there are two central binding site communities that play the roles of the network hubs of smaller peripheral communities. The sizes of communities follow a power-law distribution, which indicates that the binding sites included in larger communities may be older and have been evolutionary structural scaffolds of more recent ones. Structurally similar binding sites in the same community bind to diverse ligands promiscuously and they are also embedded in diverse domain structures. Understanding the general principles of binding site interplay will pave the way for improved drug design and protein design.

## Introduction

Ligand binding sites are responsible for various biological processes of proteins such as signal transduction and enzymatic activity. Understanding the characteristics of binding sites is essential in drug discovery and protein engineering. Still, there are many open questions that should be addressed to better understand binding sites. Are there common motifs of binding site structures? How much are binding sites similar or different quantitatively? How do binding sites evolve? Do they follow the same or different evolutionary pathways from protein domain structures? How do binding site similarities relate to known global structural, that is, fold similarities? Exploiting the vast structural data available in the Protein Data Bank (PDB)^1^, many binding site structure comparison algorithms and databases were developed^2–10^. However, previous studies were mainly focused on finding individual similar structures, and did not explore the global organization of binding site similarities^4, 6–9^. There have been studies to find clusters of known binding sites^6, 7, 11–16^. However, most of them focused on certain protein families and did not address the global and evolutionary relationships between the representative binding site structures.

In contrast to binding site structures, protein domains have been investigated extensively since domains have long been considered as basic protein structural units that are stable, function and evolve^17^. There are many approaches to classify the protein domain universe systematically, such as SCOP^18^ and CATH^19^, that provide global hierarchical organizations of domain structures. To visualize the complex global relationships between domains the whole protein domain space has been projected onto two- and three-dimensional maps^20–23^. Alternatively, the domain space has been represented as a network in which nodes are protein domains, and connections are drawn between domains that have similar folds^24, 25^. The three-dimensional projection of the domain space shows that all domain structures can be clustered into four clusters, which approximately correspond to the four SCOP classes, i.e., all alpha, all beta, alpha + beta, and alpha/beta classes^20–22^. Another study proposes that the alpha/beta class forms a densely populated and functionally diverse core region of the protein domain universe^23^. A recent network-based approach shows that alpha/beta domains form a large connected domain network, whereas all-alpha, all-beta, and alpha + beta domains form smaller and disconnected networks^24^.

It is still an open question whether the domain is the optimal level to describe and classify protein structures^26^. Several studies suggest the existence of highly conserved and frequently occurring subdomain level motifs^27–30^. Assuming that subdomain level local structures, such as binding sites, are the basic units of protein structure evolution, additional questions can be asked. What is the process by which the binding sites evolve and how does this process differ from the evolution of domain structures? Although convergent^31^ and divergent^32^ evolution of binding sites has been described, a mechanism that can encompass both evolutionary pathways has not been found yet. To help find answers to these questions, we analyzed global relationships between domains and binding sites.

In this study, we generated a global weighted network of all known binding sites based on their structural similarities calculated using ProBiS^2–4, 10^. Since the generated networks have large numbers of nodes and edges, they are incomprehensible at first glance. To reveal the hidden global organization of the networks, we reduced its complexity using a community detection approach that finds a subset of nodes that are more densely connected than the rest of the network. This approach, used in a protein-protein network improves the accuracy of protein function prediction^33–35^.

We found that the size distribution of binding site communities follow a power-law distribution, implying that the largest communities may be the motifs of the most ancient binding sites and they many have served as structural scaffolds for other binding sites. We also found that functional diversity of proteins is independent of the binding site community size, indicating that binding sites are tightly coupled to protein function. We believe that the global binding site network communities can contribute to development of new approaches to function prediction, drug discoveries, and binding site design.

## Results and Discussion

**Figure 1:**
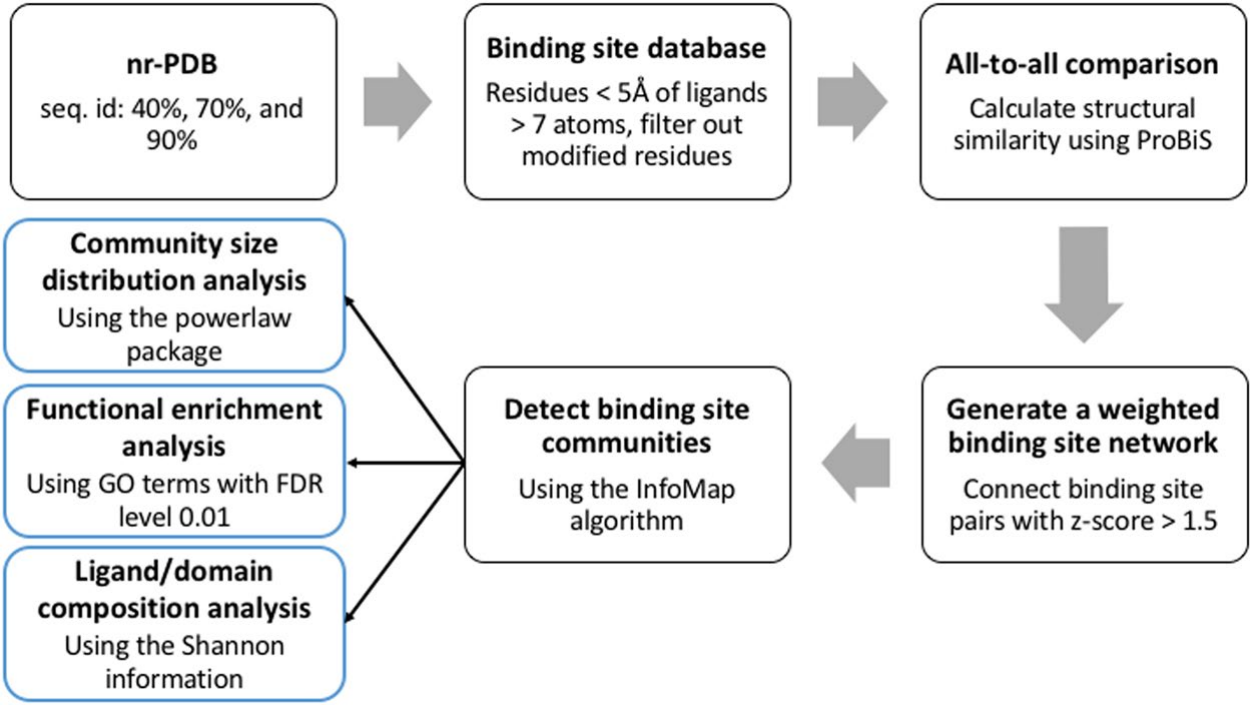
Flow diagram for binding site community analysis.

We generated a weighted binding site network from an all-to-all similarity comparison of binding site structures extracted from non-redundant sets of protein structures. In the binding site network, nodes are binding site structures and similar sites are connected by edges. Figure 1 presents the workflow in a sequence of 5 steps: 1) we generated the non-redundant protein structure databases using sequence identity cutoffs of 40, 70, and 90%; 2) non-redundant binding site structures were extracted from the databases; 3) structural similarities between all binding sites were calculated using the ProBiS^2, 3^; 4) the similarity scores were normalized into z-scores and the pairs of binding site pairs whose z-score is higher than a pre-defined threshold were connected by edges and 5) structurally similar binding sites were classified into communities to reduce the complexity of the network. Using the identified binding site communities, we investigated their size distribution, functional enrichment, and their relationships with ligands and domain structures.

### Binding site network is continuous

To identify the continuity of the binding site network, we constructed networks with z-score thresholds from 1.5 to 4.0 with an interval of 0.5, calculated the fraction of binding sites included in the largest connected network and counted the number of separate networks (Fig. S1). We identified that most binding sites are connected as a single continuous network until z-score reaches 2.5. At this z-score, only the 3% highest similarities are considered and 53% of the nodes belong to the largest connected network. This suggests that binding site structure space is continuous until this level of statistical significance. When more stringent z-scores are used, the network becomes scattered into small and disconnected networks. When z-score is 3.0, the largest connected network includes only 9.5% of the nodes and all binding sites are divided into 1,176 separate networks. For further analysis, we used the largest connected network generated with a sequence identity cutoff of 70% and a z-score threshold of 1.5, which contains 15,789 binding sites (99.8% of all non-redundant binding sites) and 547,305 edges.

### Community structure of the binding-site network reveals the global structure of binding sites

**Figure 2:**
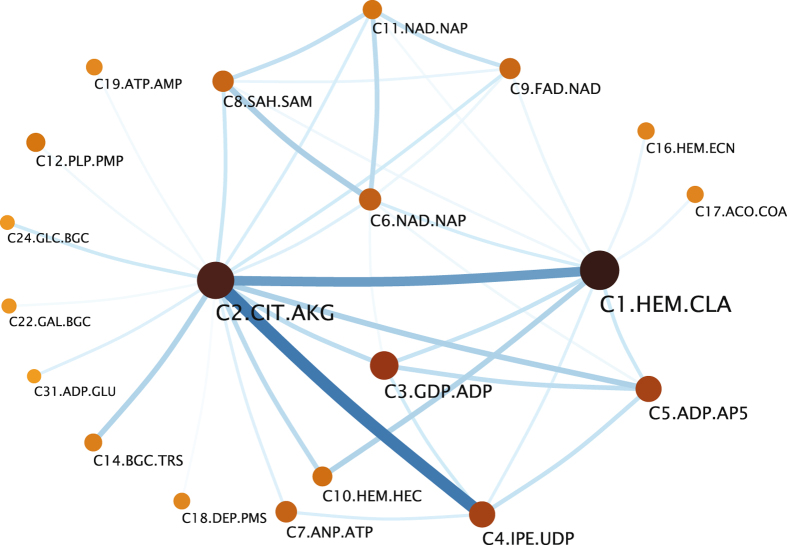
Binding site community network. The 39 highest similarities between binding site communities and associated 20 binding site communities using ProBiS are displayed. A node corresponds to a binding site community and its size is proportional to the number of included binding sites and the bigger nodes correspond to higher ranked communities. Node shade represents the aggregated structural similarity between binding sites in the community. Edge width is proportional to the structural similarities between communities. Node label, e g., C1.HEM.CLA, is composed of the community rank (C1 is the community of rank one) according to the number of the included binding sites, and of the PDB codes of the two most populated ligands (HEM stands for heme, CLA is chloropyll a). The binding site communities shown in this network contain 43.3% of all non-redundant existing binding sites in the PDB database. The ligand IDs associated with binding site communities from C1 to C10 are listed as follows: CIT – citric acid, AKG – alpha-ketoglutaric acid, CLA – chloropyll a, HEM – heme, GDP – guanosine-5′-diphosphate, ADP - adenosine-5′-diphosphate, IPE – isopentenyl pyrophosphate, POP – pyrophosphate 2^−^, AP5 - bis(adenosine)-5′-pentaphosphate, NAD - nicotinamide adenine dinucleotide, NAP - nicotinamide adenine dinucleotide phosphate, ANP - phosphoaminophosphonic acid-adenylate ester, ATP - adenosine-5′-triphosphate, SAH - S-adenosyl-L-homocysteine, SAM - S-adenosylmethionine, FAD - flavin adenine dinucleotide, HEC – heme C. The full list of community detection results as well as the rest of ligand IDs and their associated names are listed in Supplementary Information.

To reduce the complexity of the network, we detected the binding sites communities, i.e., groups of binding sites that are more closely related to each other than to the rest, using the Infomap algorithm^36, 37^. This procedure leads to the weighted network, in which nodes correspond to binding site communities and the widths of edges represent their structural similarity (Fig. 2). This community network reveals that larger communities are located at the core region of the network and smaller communities are at peripheral region. The two largest communities (C1.HEM.CLA and C2.CIT.AKG, which are most enriched with citric acid and heme molecules) are connected with most communities indicating that they play roles of network hubs. The largest communities show significant structural similarities between themselves. The 30 highest edge weights are observed between the 31 largest communities (Fig. S2). Only a few significant inter-community similarities are not connected with C1.HEM.CLA or C2.CIT.AKG.

The network of binding site communities reveals the relationships between binding sites. It is noticeable that C2.CIT.AKG has a strong connection with C4.IPE.UDP and many connections with other communities, indicating that the structure of C2.CIT.AKG may be close to the structural scaffold of many binding sites. It is also identified that a group of four communities, which interact with ligands containing adenosine, C6.NAD.NAP, C9.FAD.NAD, C8.SAH.SAM, and C11.NAD.NAP, are closely inter-connected. This suggests that these communities may be structurally diversified due to functional reasons although they interact with similar ligands. Thus, comparing the difference between these communities may provide insight into how protein structures have evolved to make similar ligands interact with various proteins in a distinctive way.

To test the robustness of our findings, we generated another binding site network using a different binding site comparison program G-LoSA^38^ and performed the community detection on the network (Fig. S3). The largest binding site community is most enriched with heme molecules. It is also identical that the five largest communities are also the most strongly connected. To verify that the similarity between community structures obtained with ProBiS and G-LoSA are statistically significant, we calculated the normalized mutual information (NMI) between two community structures and compared it with the values obtained with randomly permutated communities. The NMI value of the two original community structures was 0.337 while that of the random permutations was 0.1226 ± 0.0012, resulting in a P-value < 0.0001, which demonstrates that the community structures are independent of the choice of a binding site comparison algorithm. We also iterated the identical analysis with networks constructed with a different z-value (z = 2.0) (Fig. S4) and sequence identity thresholds, 40% and 90% (Fig. S5), and obtained similar community structures to those shown in Fig. 2.

Our analysis is the first study that identified the similarities between the communities of binding sites compared to previous PDB-wide binding site analyses^13, 14^. The most recent comprehensive global comparison of binding sites was reported by Gao and Skolnick^13^. They performed a PDB-wide clustering of about 20,000 non-redundant binding site structures using the APoc method^8^, and suggested that all binding site structures may be categorized into around 1,000 shapes. The APoc method normalizes a similarity score based on the size of a query binding-site structure, which makes the score asymmetric and dependent on a query-template definition. Thus, only the pairs that show significant similarities in both directions are used, i.e., only binding sites with similar sizes are considered, leading to tightly inter-connected communities. Similarly, Kinjo and Nakamura also performed a global similarity analysis of binding sites^14^. By performing all-to-all similarity comparison of 180,000 binding sites known by June 2008, they constructed 11,532 separate networks consisting of highly similar binding sites both functionally and structurally. The large number of separated networks is due to the use of an exceedingly stringent similarity criterion to connect binding sites (P-value < 10^−15^). In contrast to previous studies, we used the z-score of the ProBiS scoring function^4^, which normalizes a similarity score based on the number of aligned residues, resulting in scores independent of the binding site size. Thus, the binding site communities detected in this study consist of more remotely and weakly related binding sites. In addition, our network reveals relative similarities between binding site communities, which may shed light on the evolution of binding site structures.

### Sizes of binding site communities follow a power-law distribution

**Figure 3:**
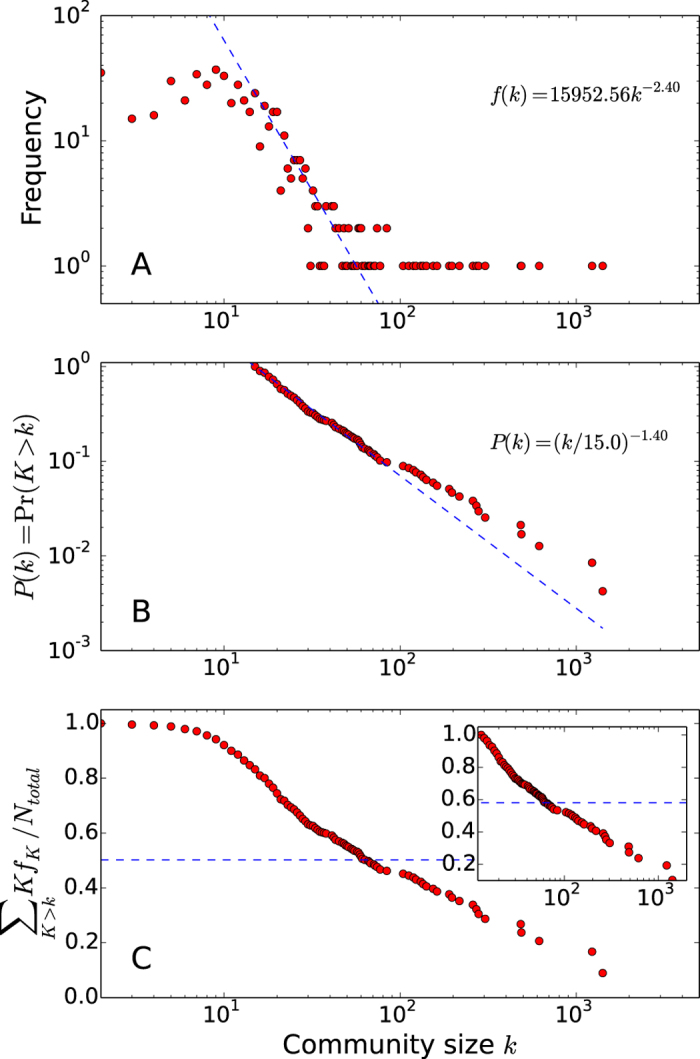
Size distributions of binding site communities. (**A**) The frequency of binding site communities of size k, (**B**) the complementary cumulative distribution function (cdf) of community sizes *P*(*k*), and (**C**) the cumulative fraction of binding sites included in binding site communities whose sizes are larger than k are plotted. The cdf function is plotted using the minimum community size of 15, which is determined by the power-law fitting. The inset of the plot (**C**) shows the cumulative fraction of binding sites included in the communities with more than 14 binding sites. *N*_*total*_ is the total number of binding sites in the network. The blue dotted lines in (**C**) represent the cumulative fractions included in the 30 largest communities. When all communities are considered, 50% of sites are included in the 30 largest communities. If only the communities larger than 14 are considered, 58% of binding sites are included.

The size distribution of binding site communities follows a power-law distribution. The linearity of the complementary cumulative distribution function^39^ clearly shows that the community sizes are distributed by a power-law: *f*(*k*) = 15953*k* ^−2.4^, where *f*(*k*) is the number of communities with a size of *k* (Fig. 3A and B). The power law distribution of the communities indicates that a few extremely large communities coexist with many small ones. The 30 communities shown in Fig. 2 include 47.7% of all binding sites (Fig. 3C), thus, a few largest communities and their direct neighbors represent the majority of them. The generative models explaining the power-law distribution are generally based on the constant birth rate assumption, i.e., the probabilities to be duplicated or emerge a new community are identical for all binding sites regardless of their structure or function^40–43^. The only dominant factor that determines the future community size is its current size. Thus, the power-law distribution suggests that binding sites may have evolved based on a simple and universal mechanism, not constrained by a structural or functional necessity^44, 45^.

The power-law distribution of the binding site communities also implies that the largest communities may be the most ancient binding site structures and they might have been structural scaffolds for the evolution of other binding sites. A power-law distribution is widely found in genomics^44, 46^ and it has been successfully explained by simple generative models^41, 44^, which indicate that a larger community is generally older than a smaller one. The analyses of protein domain structures show that alpha/beta domains are located at denser regions, i.e., have more structural neighbors, and, generally, are older than other folds^22–24^. To further test this hypothesis, we calculated the enrichment of taxonomies of binding sites^47^. If the hypothesis is valid, binding sites from older organisms should be mostly found in large communities. Our analysis indicates this is the case. Binding site structures from cyanobacteria are enriched in only the first two largest communities (Table S5). C1.HEM.CLA is enriched with a DNA binding and cytochrome-c oxidase activity and C2.CIT.AKG is highly enriched with transition metal ion (Zn^2+^, Fe^2+^, Fe^3+^, Mn^2+^ and Co^2+^) binding functions. This is consistent with a domain-based proteome study^48^ and phylogenetic analyses^49, 50^. Whereas binding sites from human proteins are enriched not only in large communities (C3.GDP.ADP and C7.ANP.ATP) but also in many small communities (C13, C51, C52, C90, C100, C183, C189, and C241) (Table S6). Interestingly, C3.GDP.ADP is highly enriched with ATPase activity and an ABC transporter function. This fact is consistent with the recent domain structure analysis, which found that the ABC transporters might be the oldest aerobic metabolic enzymes^51^. Thus, the taxonomy analysis also supports the hypothesis that larger communities may be more ancient and have been evolutionary structural scaffolds of smaller ones.

The binding sites that belong to small communities with less than 15 binding sites, which do not follow the power-law distribution mainly bind to glycans or buffering agents. The most frequently found ligand in these small communities is N-acetyl-D-glucosamine (NAG), which is one of the most common building blocks of glycans. Because N-glycosylation process involves the formation of a covalent bond between a glycan and the residue at a glycosylation site, NAG binding site residues may play little role in its binding, which makes these residues non-specific binding partners. Following NAG, 2-methyl-2,4-pentanediol (MPD) and 2-(N-morpholino) ethanesulfonic acid (MES) are the next most frequently found ligands in the small communities; these are used as buffering or precipitation agents. This suggests that the binding sites in the small communities may not be true binding sites and the power-law fitting may allow us to discriminate between specific and non-specific interactions.

### Binding site communities are functionally specific

To study the relationships between binding site structures and functions and to functionally characterize each community, we performed the functional enrichment analysis by mapping the gene ontology (GO) annotations^52^ onto communities. We found that the largest binding site communities are associated with different functions and have few overlapped functions despite their significant structural similarities. The enriched functions of C1.HEM.CLA detected with a P-value threshold of 0.001 are compared with those of the other top 30 largest communities. Among them, only 9 communities have at least one common functional annotation. Further, the all-to-all comparison between the top 30 communities showed that the average number of shared functional annotation is only 0.47. These results indicate that a binding-site structure is strongly coupled with the function of a protein and our binding-site centric classification of proteins is thus very function specific.

For example, the two tightly connected communities C6.NAD.NAP and C9.FAD.NAD have NAD as their major ligands and are enriched with oxidoreductase activity related terms. However, C6.NAD.NAP is enriched with NADH-specific enoyl-ACP reductase activity (GO:0004318) while C9 is enriched with dihydrolipoyl dehydrogenase activity (GO:0004791). Overall, the two communities share only 4 enriched GO terms although C6 and C9 have 24 and 18 enriched terms, respectively. This indicates that our community analysis also differentiates the binding sites that bind to the same ligand but have different functions.

**Figure 4:**
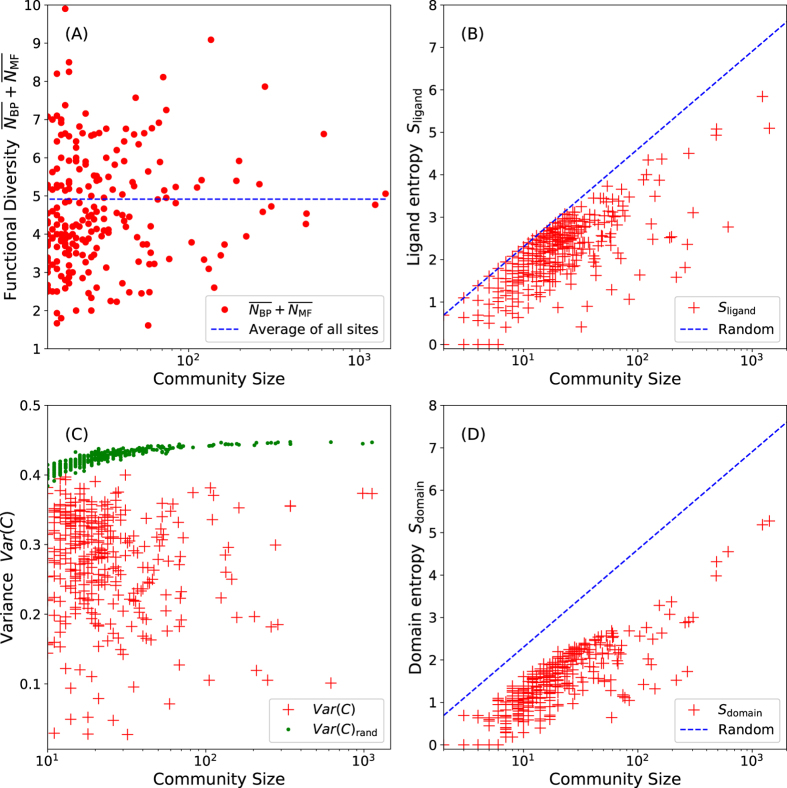
Shannon information (entropy) values of the ligand/domain compositions and the functional diversity of binding site communities The x-axes represent the community size using a log-scale. The y-axis of (**A**) represents the functional diversity of the communities. The average functional diversity of a community is measured by the average number of distinct GO-BP ($\overline{{{\boldsymbol{N}}}_{{\rm{BP}}}}$) and GO-MF ($\overline{{{\boldsymbol{N}}}_{{\rm{MF}}}}$) terms of included proteins. The average functional diversity of all proteins in the network, 4.9, is denoted as the blue dotted line. The y-axes of subplot (**B**) and (**D**) represent the Shannon information values of ligand and domain compositions of communities. The Shannon information values were calculated as follows: ${\boldsymbol{S}}=-\sum _{{\boldsymbol{i}}}{{\boldsymbol{p}}}_{{\boldsymbol{i}}}\,\mathrm{ln}\,{{\boldsymbol{p}}}_{{\boldsymbol{i}}}$, where *i* is the ligand or the domain index. The y-axis of subplot (**C**) represents the variance of the distances between ligands in a community: ${\rm{Var}}({\boldsymbol{C}})=\frac{1}{{{\boldsymbol{n}}}^{2}}{\sum }_{i}{\sum }_{{\boldsymbol{j}} > {\boldsymbol{i}}}{(1-{{\boldsymbol{T}}}_{{\boldsymbol{ij}}})}^{2}$, where *T*_*ij*_ is the Tanimoto coefficient^53^ between ligands i and j. The variances of the binding sites communities are plotted with red crosses and the green dots correspond to the variances of the same number of randomly selected ligands.

Next, we investigated the functional diversity of proteins associated with binding site communities. It is known that the domain structures located at the core region of the protein structures map tend to have more diverse functions than the proteins at peripheral regions^23^. We measured the functional diversity by obtaining the average number of distinct GO molecular function (*N*_MF_) and biological process (*N*_BP_) terms associated with each protein included in each community^23^. Our results show a different pattern from the results of domain structure analysis; there is little correlation between the centrality of a binding site community and the functional diversity of its members (Fig. 4A). The functional diversity of proteins is almost uniform regardless of its community size, and is 4.9, which is the average functional diversity of all proteins in the network.

### Binding site communities are promiscuous

Do similar binding sites bind to similar ligands? To quantify the ligand specificity of binding site communities, we calculated the ligand composition Shannon information *S* (entropy) of a binding site community. The entropy is calculated using the following formula: ${\boldsymbol{S}}=-\sum _{{\boldsymbol{i}}}\,{{\boldsymbol{p}}}_{{\boldsymbol{i}}}\,\mathrm{ln}\,{{\boldsymbol{p}}}_{{\boldsymbol{i}}}$, where *i* is a ligand or a domain index and *p*_*i*_ is the fraction of ligand in a community. A large value of *S* indicates that a binding site in the community binds to diverse ligands, and *S* close to zero, indicates that it binds to a few specific ligands. We found an almost linear relationship between the size and the ligand composition Shannon information of the binding site communities (Fig. 4B). The linear relationship is close to the theoretical maximum Shannon information when all ligands are different, i.e., S = log *N*_*comm*_, where *N*_*comm*_ is the size of a community. We also calculated the variance of distances between ligands in a community to identify whether the same relationship is valid when the chemical similarities of ligands are considered (Fig. 4C). The variances of ligand similarities of binding site communities are compared with those of the same number of randomly selected ligands. A lower variance indicates that the included ligands are more similar to each other. The analysis shows that the majority of detected communities have large variances approaching random distribution. On the other hand, only a few communities have significantly lower variances than randomly selected sets, e.g., eleven communities have variances lower than 0.1. These results indicate that similar binding sites included in the same community bind to diverse ligands and the diversity of ligands increases with the size of communities, which is consistent with previous studies^13, 54^.

This high ligand promiscuity of binding site communities may be explained by the constructive neutral evolution scenario^44, 45, 55^, in which a system evolves via the accumulation of irreversible dependencies between related parts of the system, not adaptation. The scenario is known to be consistent with the power-law distribution of community^44, 45^. If binding sites in larger communities are more ancient than ones in smaller communities, more diverse ligands could have been tested against them, which may have resulted in more binding partners than recently evolved sites.

### Similar binding sites are found in diverse domain structures

Are similar binding sites embedded in similar domain structures? To answer this question, we calculated domain composition Shannon information *S* values by investigating to which CATH domain a binding site belongs. Overall, the domain composition *S*_domain_ of a binding site community increases almost linearly with the logarithm of its size, similar to the ligand composition *S*_ligand_ (Fig. 4D). In other words, a binding site structure from a larger community is associated with more diverse backbone structures. However, *S*_domain_ values deviate more from the theoretical maximum compared to *S*_ligand_ values, which indicates that a binding site structure depends weakly on its domain structure.

The fact that binding site structures depend only weakly on their corresponding domain structures indicates that the protein structure segments may have evolved independently. In other words, the unit of protein structure evolution could be smaller than a domain^27–29, 56^. If a binding site and its backbone structure were strongly coupled, *S*_domain_ should be significantly lower than the theoretical maximum and almost completely independent of the community size. Halabi *et al*.^29^ suggest the concept of protein sectors, in which a whole domain can be divided into the subgroups of residues that are structurally adjacent and have distinct functional roles. They show that the S1A serine protease domain consists of three functionally independent groups of connected residues that have the roles of ligand specificity, protein stability and catalytic core, respectively^29^. The protein sector analyses of the PDZ, PAS, SH2, and SH3 domain families show that the ligand binding sites of these domains are detected as independent sectors^29^.

The protein sector hypothesis could explain the differences between the functional diversity distributions of the domain network and our binding site network. It was identified that the core of the domain network, mostly alpha/beta classes of SCOP, has high functional diversity, and the peripheral region has low functional density^23, 24^. However, the functional diversity of binding sites is almost uniform across the binding site network, which suggests that binding sites are more important in determining the function of a protein than its domain structure. If a domain structure consists of independent sectors, the combinations of functionally distinct sectors can achieve high functional diversity of a domain. A binding site that recognizes a specific ligand can form a domain with various sectors performing distinct enzymatic activities, and the sectors in the domain communicate via an allosteric mechanism. In contrast, the domains with lower functional diversity may consist of sectors having similar functions.

### Perspective

It is reasonable to propose that the global binding site network and detecting its community structure can contribute to development of new methods for structure-based function inference, drug discovery, and binding site design. High functional specificity of binding site communities proposes that more accurate protein function predictions will be possible using the knowledge on similar binding sites and the enriched functions of the communities than by using protein domain structures^34, 35^. The enhanced prediction accuracy may be more pronounced when a target protein adopts an alpha/beta structure, since this fold has high functional promiscuity^23^, which can lead to many false positive predictions. Also, knowing which binding residues are conserved in a community may significantly improve the success rate of drug discovery and protein design.

Our analysis identified that interactions between ligands and binding pockets are generally promiscuous. This explains the “off-target” effects of drugs because one specific ligand may bind to different binding pockets^13, 57^. This also suggests that a conventional strategy to find new drug candidates that considers the nearest neighbors of a specific ligand may be inefficient. Although binding site-ligand interactions are generally promiscuous, our analysis also indicates that different binding site communities are functionally distinct. Based on this observation, one alternative approach to overcome the limitation of promiscuous interaction is to extract useful information from the ensemble of related binding sites and their ligands. For example, certain interactions between a given ligand and related binding sites in a community may be conserved, while no such interactions exist between the same ligand and binding sites in another community. If this is the case, such difference may be used to screen or design new more selective drug candidates. Similar approaches, using an ensemble of related ligand-binding site interactions or considering remote homologs of a target binding site, have been suggested previously^58–63^. We believe that the network of binding site communities will provide a basis for exploring more efficient computational approaches for drug discovery and design.

## Materials and Methods

### ProBiS algorithm

To define surface residues, solvent accessible area was defined by rolling a spherical probe of a radius of 1.4 Å on the van der Waals surface of protein atoms. Residues located up to 4 Å below this surface were considered as surface residues. Binding site residues were determined as those surface residues separated by <5 Å from any ligand atom. Ligands were defined as those HET codes in the PDB file having >7 heavy atoms, and not being a modified residue denoted by a MODRES code. Thus, surface binding sites as well as binding sites deep inside the proteins that are connected with the exterior by a channel with radius of at least 1.4 Å were considered. Ligand binding sites that are completely buried within a single protein chain are very rare in our experience and were not considered in this study; nevertheless, binding sites buried by two or more chains were still considered, as each protein chain was considered separately. Metal ions are not considered as ligands in this study. Nevertheless, metal ion binding sites are considered if they are in vicinity (<5 Å) of a small molecule ligand.

A detected binding site surface patch was represented as a graph consisting of a set of vertices and edges connecting them. Vertices in a graph represent the functional groups of surface amino acid residues. A functional group is characterized with 5 groups based on its physicochemical property: hydrogen bond donor, acceptor, mixed donor/acceptor, aromatic, and aliphatic^64^. If two vertices are separated by <15 Å, they are connected by an edge.

For a given pair of binding site graphs, their product graph was constructed. For two graphs, *G*_1_ and *G*_2_, the vertex set of the Cartesian product graph is defined as: $H=V({G}_{1})\times V({G}_{2})=\{(u,v)|u\in V({G}_{1})\,{\rm{and}}\,v\in ({G}_{2})\}$. If the physicochemical properties of two vertices of the initial graphs, *u* and *v*, are different, the corresponding pair is not considered to generate the product graph. Two vertices of a product graph, (*u*_1_,*v*_1_) and (*u*_2_,*v*_2_), are connected by an edge if and only if the distances between *u*_1_ and *v*_1_ and between *u*_2_ and *v*_2_ differ by <2 Å. The generated product graph is an approximate representation of all possible superposition of two structures. From the generated product graph, we detected its maximum clique^2, 65^, i.e., the largest complete sub-graph of a graph where all vertices are connected to each other.

For each pair of aligned binding sites, we calculated its similarity score using structural similarity and evolutionary similarity^4^. For each pair of binding site alignment, four criteria were used to calculate the local alignment score: (1) surface angle, (2) surface patch RMSD, (3) surface patch size, and (4) an E-value calculated with the Karlin-Altschul equation^66^. For each surface patch, a surface vector originating from the geometric center of the patch and pointing to the perpendicular direction of the surface was generated. If the angle between a pair of surface vectors was larger than 90° or the number of aligned vertices were smaller than 10, the pair was discarded. For the remaining pairs, the alignment scores, *al*_*score*_, were calculated as follows: $a{l}_{{\rm{score}}}=\,\mathrm{log}(\frac{{n}_{{\rm{vert}}}\times \,\mathrm{log}(1+1/{e}_{{\rm{value}}})}{RMSD})$, where RMSD is the surface patch RMSD between pairs of superimposed vertices, *n*_*vert*_ is the number of aligned vertices, and *e*_value_ is the alignment expectation value^4^. The raw alignment scores were normalized to z-scores as follows: $z-{\rm{score}}=\frac{a{l}_{{\rm{score}}}-\mu }{\sigma }$, where μ and σ are the average and the standard deviation of the alignment scores of all pairs, and the values of μ and σ are 2.0 and 2.2, respectively.

### Constructing a binding site network and detecting communities

We constructed an initial non-redundant protein single chain database from a Nov 2013 PDB release^1^ using a sequence identify cutoff of 95% resulting in 42,282 unique protein chains. Based on the non-redundant protein chain database, an all-to-all comparison between the surfaces of the non-redundant chains was performed using ProBiS^2–4^. All details on the ProBiS algorithm are reported in SI Methods. From the statistics of similarity scores from the all-to-all comparison, we calculated the z-scores of all similarity scores. A pair of binding sites is connected by an edge if the z-score of the pair is higher than a threshold value and the z-score of the edge is represented by its weight. If the z-score of a pair is lower than a threshold, the pair is considered to be disconnected. The G-LoSA method was used on the same binding site set. A community detection analysis was performed using the Infomap method based on the minimum description length principle^36, 37^. This method detects the community structure of a network by minimizing the amount of information to describe the itinerary of a random walker on the network. One of the advantages of the Infomap method is that it yields how strongly communities are related, which is interpreted as the similarity between a pair of binding site communities in this study. The weights of inter-community edges are proportional to the probability of a random walker selecting the path. A higher inter-community probability indicates higher structural similarity between binding site communities.

### Power-law fitting

We fitted the size distribution of binding site communities to a power-law distribution using the powerlaw Python package^67^. The optimal minimum threshold of the cluster size for fitting was determined by the algorithm to yield the minimum Kolmogorov-Smirnov distance between the data and the fit^68^. The sizes of communities and their frequencies are fitted to a power-law distribution, $p(k)=\Pr (K=k)=\,C{k}^{-\alpha }$, where Pr(K) is the probability and *p*(*k*) probability density to form a community with *k* binding sites; *α* is a scaling parameter and *C* is a normalization constant. The fitting showed that the communities with more than 14 binding sites, *k*_*min*_ = 15, follow a power-law distribution with *α* = 2.40^67, 68^. The total number of communities with more than 14 sites is 236. The most common way to identify a power-law distribution is to check the linearity of the cumulative distribution (cdf), *P*(*k*) = Pr(K > *k*), on a log-log plot^68^.

### Ligand and domain composition Shannon information calculation

The ligand and domain composition Shannon information values were calculated using the following formula: $S=-{\sum }_{i}{p}_{i}\,\mathrm{ln}\,{p}_{i}$, where *i* is a ligand or a domain index and *p*_*i*_ is the fraction of ligand or domain *i* relative to the total number of binding sites in a community. For ligand composition Shannon information calculation, we used the ligand IDs defined in the PDB database. Ligands are considered as different if they have different ligand IDs in the PDB. To identify the promiscuity of a binding site community, we also calculated the variance of ligand similarities. The variance of ligands in a community *C* is calculated with the following equation: ${\rm{Var}}(C)=\frac{1}{{n}^{2}}{\sum }_{i}{\sum }_{j > i}{(1-{T}_{ij})}^{2}$, where *T*_*ij*_ is the Tanimoto coefficient^53^ between ligands *i* and *j*.

For domain composition Shannon information calculation, the chain IDs of residues participating in forming a binding site were identified and their domain IDs defined by the CATH database^19, 69^ were used. If a binding site consists of residues from multiple distinct chains, the binding site was considered to be located at the interface of those chains. A binding site located at the interface of a homo-oligomer was considered to be different from that of the corresponding monomer.

### Functional enrichment analysis

To identify which functional annotations are significantly enriched in a community, the P-values of GO terms^52^ associated with proteins included in a community were calculated. In this study, we used the MF and BP terms of GO annotations. P-values were calculated using a hypergeometric distribution:

$$P=1-\sum _{i=0}^{k-1}\frac{(\begin{array}{c}f\\ i\end{array})(\begin{array}{c}n-f\\ m-i\end{array})}{(\begin{array}{c}n\\ m\end{array})},$$

where *n* is the total number of proteins in the network with known GO terms, *m* is the size of a community, *f* is the total number of proteins in the network associated with a function of interest, and *k* is the number of proteins in the community with the function of interest.

In other words, the proteins in the network with known associated GO terms were used as the background protein list. The GO annotations of the proteins were adopted from the SIFTS database^47^. Since GO terms are hierarchical, only the most specific terms of each protein were used^23^. For each community, P-values were adjusted for multiple comparisons using the Benjamini-Hochberg procedure with a false discovery rate level of 0.01^70^, indicating that less than 1% of identified enriched functional annotations are expected to be false positives.

### Data availability

All relevant data are available from the authors upon request.

## Electronic supplementary material

Supplementary Information

Dataset 1

Dataset 2

Dataset 3

Dataset 4

Dataset 5

Dataset 6
