# Supplementary material for: Global organization of a binding site network gives insight into evolution and structure-function relationships of proteins
Source: Sci Rep. 2017 Sep 14;7:11652. doi: 10.1038/s41598-017-10412-z (PMC5599562; doi:10.1038/s41598-017-10412-z)
Supplement: Supplementary file 1 — Supplementary Information [file 41598_2017_10412_MOESM1_ESM.pdf]

Supporting Information

Global organization of a binding site network gives insight into evolution and structure-function relationships of proteins

Juyong Lee[1,*,†], Janez Konc[2,3,†], Dušanka Janežič[2], and Bernard R. Brooks[1]

[1]Laboratory of Computational Biology, National Heart, Lung, and Blood Institute, National Institutes of Health, Bethesda, Maryland 20892, United States

[2]Faculty of Mathematics, Natural Sciences and Information Technologies, University of Primorska, Glagoljaška 8, SI-6000, Koper, Slovenia

[3]National Institute of Chemistry, Hajdrihova 19, SI-1000 Ljubljana, Slovenia

[*]Correspondence to juyong.lee@nih.gov
†These authors contributed equally to this work.

Supplementary figures and tables

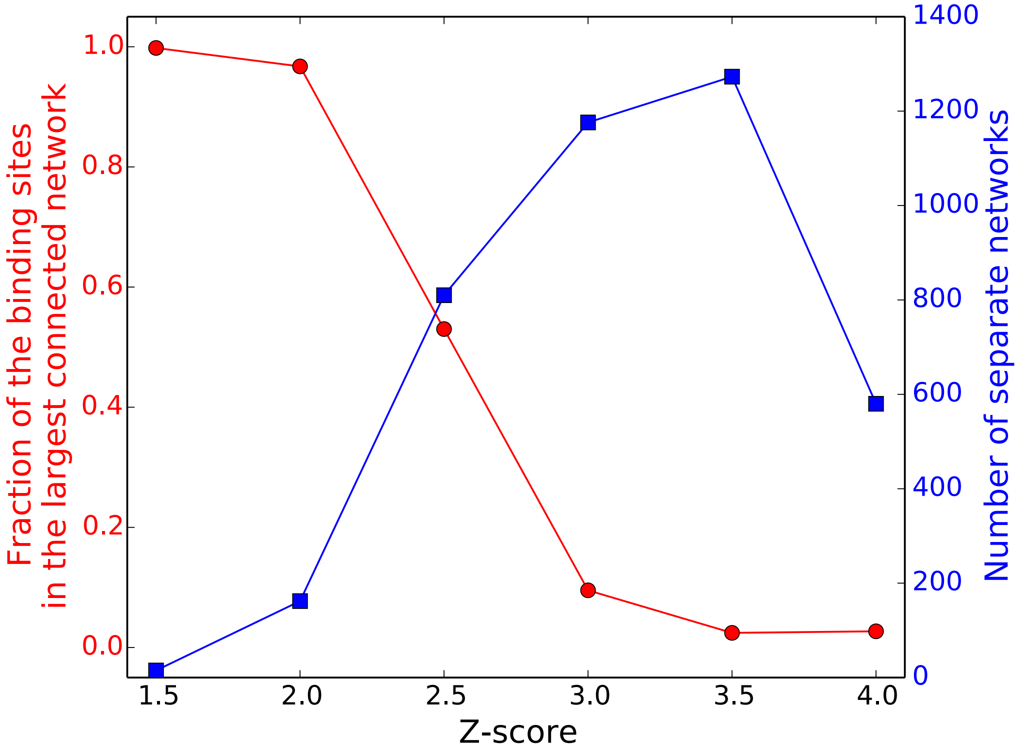

Figure S1 Binding site continuity analysis. The fraction of the binding sites included in the largest component (left, red) and the number of disconnected networks along different z-value thresholds (right, blue) are displayed.

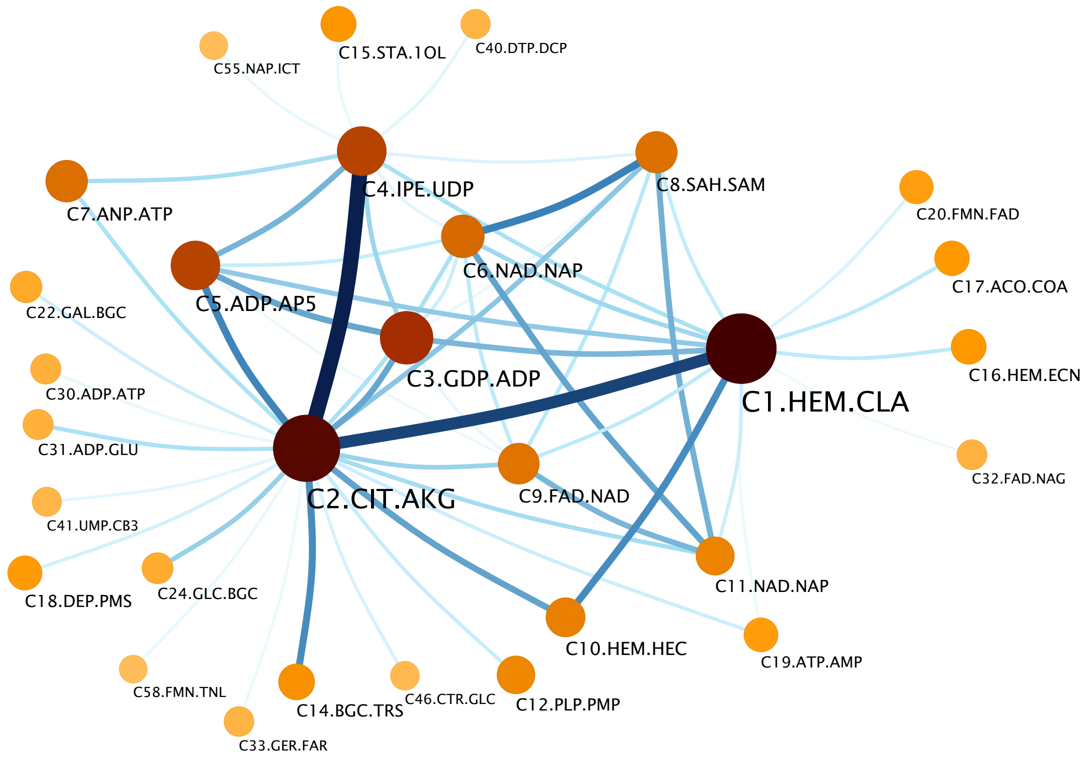

Figure S2 Binding site community network. The 55 highest similarities between binding site communities and associated 30 binding site communities using ProBiS are displayed. A node corresponds to a binding site community and its size is proportional to the number of included binding sites and the bigger nodes correspond to higher ranked communities. Node shade represents the aggregated structural similarity between binding sites in the community. Edge width is proportional to the structural similarities between communities. Node label, e g., C1.HEM.CLA, is composed of the community rank (C1 is the community of rank one) according to the number of the included binding sites, and of the PDB codes of the two most populated ligands (HEM stands for heme, CLA is chloropyll a). The binding site communities shown in this network contain 47.7% of all non-redundant existing binding sites in the PDB database. The ligand IDs associated with binding site communities from C1 to C10 are listed as follows: CIT – citric acid, AKG – alpha-ketoglutaric acid, CLA – chloropyll a, HEM – heme, GDP – guanosine-5'-diphosphate, ADP - adenosine-5'-diphosphate, IPE – isopentenyl pyrophosphate, POP – pyrophosphate 2^{-}, AP5 - bis(adenosine)-5'-pentaphosphate, NAD - nicotinamide adenine dinucleotide, NAP - nicotinamide adenine dinucleotide phosphate, ANP - phosphoaminophosphonic acid-adenylate ester, ATP - adenosine-5'-triphosphate, SAH - S-adenosyl-L-homocysteine, SAM - S-adenosylmethionine, FAD - flavin adenine dinucleotide, HEC – heme C. The full list of community detection results as well as the rest of ligand IDs and their associated names are listed in Supplementary Information.

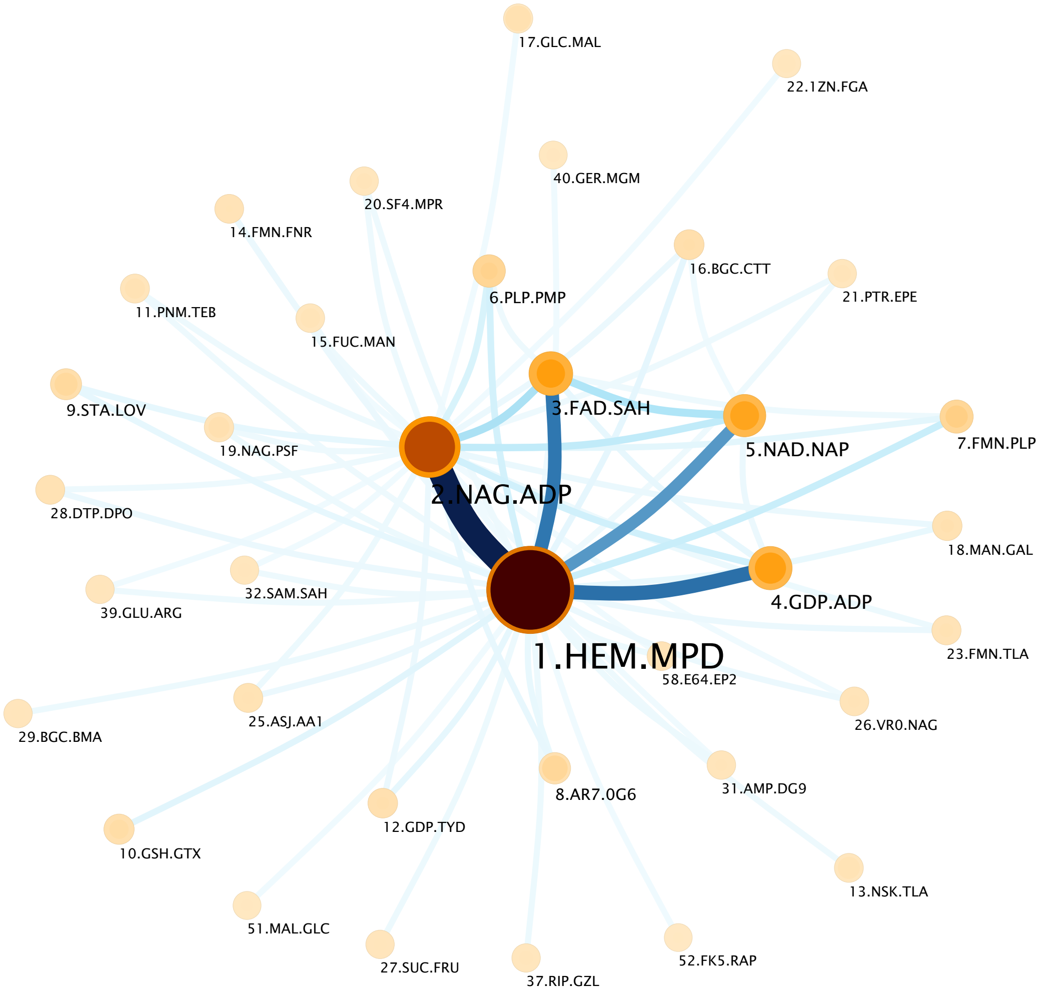

Figure S3 Binding site network generated with G-LoSA. The same non-redundant binding site set was used. A z-value threshold of 2.0 was used.

Table S1 A comparison of the community structures using the ProBiS result as a reference

ProBiS community index	G-LoSA community index	Number of overlapping binding sites (N_{inter})	Fraction of overlapping binding sites with respect to G-LoSA community	Cumulative fraction of ProBiS community
**1**	1	1163	16.5	82.3
**2**	1	514	7.3	41.8
**2**	2	415	10.6	75.5
**3**	4	461	89.9	74.8
**3**	1	116	1.6	93.7
**4**	1	225	3.2	46.1
**4**	2	125	3.2	71.7
**4**	26	27	69.2	77.3
**5**	1	212	3.0	43.8
**5**	2	177	4.5	80.4
**6**	5	189	40.0	62.2
**6**	12	47	82.5	77.6
**7**	2	251	6.4	90.0
**8**	3	209	37.2	77.1
**9**	3	189	33.6	73.0
**9**	1	33	0.5	85.7
**10**	1	178	2.5	82.0

Table S2 A comparison of the community structures using the G-LoSA result as a reference

G-LoSA community index	ProBiS community index	Number of overlapping binding sites (N_{inter})	Fraction of overlapping binding sites with respect to ProBiS community	Cumulative fraction of G-LoSA community
**1**	1	1163	82.3	16.5
**1**	2	514	41.8	23.8
**1**	4	225	46.1	27.0
**1**	5	212	43.8	30.0
**1**	10	178	82.0	32.6
**1**	16	131	97.0	34.4
**1**	3	116	18.8	36.1
**1**	18	107	87.0	37.6
**1**	17	98	74.8	39.0
**1**	19	93	78.2	40.3
**1**	20	80	71.4	41.5
**2**	2	415	33.7	10.6
**2**	7	251	90.0	16.9
**2**	5	177	36.6	21.5
**2**	4	125	25.6	24.6
**2**	1	117	8.3	27.6
**2**	29	62	91.2	29.2
**2**	30	59	88.1	30.7
**2**	31	56	84.8	32.1
**2**	22	53	63.1	33.5
**2**	43	42	79.2	34.5
**2**	27	41	56.9	35.6
**3**	8	209	77.1	37.2
**3**	9	189	73.0	70.8
**3**	2	13	1.1	73.1
**3**	11	12	6.1	75.3
**4**	3	461	74.8	89.9
**5**	6	189	62.2	40.0
**5**	11	112	56.9	63.8
**5**	38	30	51.7	70.1
**5**	89	16	57.1	73.5
**5**	92	12	44.4	76.1
**6**	12	140	73.7	94.0
**7**	58	28	68.3	15.8
**7**	2	16	1.3	24.9
**7**	77	15	48.4	33.3
**7**	195	14	82.4	41.2
**7**	156	11	57.9	47.5
**7**	124	10	45.5	53.1
**7**	190	10	58.8	58.8
**7**	105	7	28.0	62.7
**7**	306	7	63.6	66.7
**7**	5	6	1.2	70.1
**7**	185	6	35.3	73.4
**8**	13	120	74.1	81.6

**9**	15	91	64.5	94.8
**10**	21	71	68.3	95.9

Table S3 Most enriched functions of the 10 largest communities obtained with ProBiS

Community index	Size	Molecular function description	-log(P)
**1**	1413	oxygen binding	69.3
		oxygen transporter activity	58.9
		steroid hormone receptor activity	41.9
		heme binding	32.2
		DNA binding	29.3
**2**	1230	zinc ion binding	36.9
		metalloendopeptidase activity	25.0
		peptide deformylase activity	15.0
		ferrous iron binding	14.5
		hydrolase activity, acting on carbon-nitrogen (but not peptide) bonds	14.0
**3**	616	GTP binding	188.8
		ATP binding	150.5
		GTPase activity	118.7
		ATPase activity	47.7
		microtubule motor activity	31.2
**4**	488	magnesium ion binding	24.1
		geranyltranstransferase activity	12.1
		fructose 1,6-bisphosphate 1-phosphatase activity	9.7
		terpene synthase activity	6.8
		2-amino-4-hydroxy-6-hydroxymethyldihydropteridine diphosphokinase activity	6.8
**5**	484	adenylate kinase activity	17.8
		phosphotransferase activity, phosphate group as acceptor	15.7
		kinase activity	14.1
		cytidylate kinase activity	7.2
		pyridoxamine-phosphate oxidase activity	6.1
**6**	304	coenzyme binding	43.3
		oxidoreductase activity	38.1
		enoyl-[acyl-carrier-protein] reductase (NADH) activity	18.0
		3-oxoacyl-[acyl-carrier-protein] reductase (NADPH) activity	15.8
		UDP-glucose 4-epimerase activity	10.6
**7**	279	protein serine/threonine kinase activity	190.0
		ATP binding	162.6
		protein tyrosine kinase activity	65.4
		kinase activity	35.2
		protein kinase activity	26.1
**8**	271	methyltransferase activity	61.1
		O-methyltransferase activity	35.7
		mRNA (guanine-N7-)-methyltransferase activity	17.5
		site-specific DNA-methyltransferase (adenine-specific) activity	14.0
		N-methyltransferase activity	13.0
**9**	259	flavin adenine dinucleotide binding	114.4
		thioredoxin-disulfide reductase activity	28.1
		dihydrolipoyl dehydrogenase activity	24.3
		oxidoreductase activity	21.3
		NADP binding	10.6
**10**	217	electron carrier activity	151.8
		heme binding	150.7
		iron ion binding	79.0
		nitrite reductase (cytochrome, ammonia-forming) activity	11.0
		succinate dehydrogenase activity	3.8

Table S4 Most enriched functions of the 10 largest communities obtained with G-LoSA

Community index	Size	Molecular function description	-log(P)
**1**	7036	heme binding	75.7
		iron ion binding	53.3
		oxygen binding	27.2
		oxygen transporter activity	17.9
		steroid hormone receptor activity	15.1
**2**	3930	protein serine/threonine kinase activity	57.2
		ATP binding	20.7
		protein tyrosine kinase activity	20.5
		porin activity	10.2
		protein kinase activity	9.7
**3**	562	flavin adenine dinucleotide binding	66.3
		methyltransferase activity	46.8
		O-methyltransferase activity	25.0
		dihydrolipoyl dehydrogenase activity	13.1
		oxidoreductase activity	12.9
**4**	513	GTP binding	197.8
		GTPase activity	131.2
		ATP binding	116.0
		ATPase activity	41.0
		microtubule motor activity	38.0
**5**	472	NAD binding	72.3
		oxidoreductase activity	32.2
		NADP binding	31.9
		transferase activity, transferring acyl groups other than amino-acyl groups	26.4
		coenzyme binding	23.5
**6**	149	pyridoxal phosphate binding	213.7
		transaminase activity	47.2
		L-phenylalanine:2-oxoglutarate aminotransferase activity	20.0
		L-aspartate:2-oxoglutarate aminotransferase activity	11.4
		O-phospho-L-serine:2-oxoglutarate aminotransferase activity	8.3
**7**	177	FMN binding	27.9
		IMP dehydrogenase activity	14.0
		alanine racemase activity	13.3
		orotidine-5'-phosphate decarboxylase activity	12.8
		triose-phosphate isomerase activity	9.3
**8**	147	serine-type endopeptidase activity	59.3
		cysteine-type endopeptidase activity	5.7
**9**	96	aspartic-type endopeptidase activity	22.3
**10**	74	glutathione transferase activity	57.8
		transferase activity	19.2
		protein disulfide oxidoreductase activity	16.5
		electron carrier activity	6.7
		glutathione peroxidase activity	4.5

Table S5 Enrichment of binding sites from Cyanobacteria

Community ID	Taxonomy ID	P-value	Number of proteins	Number of proteins in the community	Description
**1**	197221	55.6379	95	71	Thermosynechococcus elongatus BP-1
**1**	146786	11.3401	16	13	Thermosynechococcus elongatus
**1**	83541	10.3521	15	12	Mastigocladus laminosus
**2**	1117	12.4927	17	14	Cyanobacteria
**2**	1126	10.9093	12	11	Microcystis aeruginosa
**2**	70799	6.5156	6	6	Nodularia spumigena

Table S6 Enrichment of binding sites from Homo sapiens

Community ID	Taxonomy ID	P-value	Number of proteins	Number of proteins in the community	Description
**3**	9606	6.1747	2326	139	Homo sapiens
**7**	9606	63.5619	2326	162	Homo sapiens
**13**	9606	4.4887	2326	36	Homo sapiens
**51**	9606	5.0112	2326	20	Homo sapiens
**52**	9606	8.0583	2326	24	Homo sapiens
**90**	9606	5.9271	2326	16	Homo sapiens
**100**	9606	7.874	2326	17	Homo sapiens
**183**	9606	6.611	2326	13	Homo sapiens
**189**	9606	4.9481	2326	11	Homo sapiens
**241**	9606	4.0936	2326	9	Homo sapiens

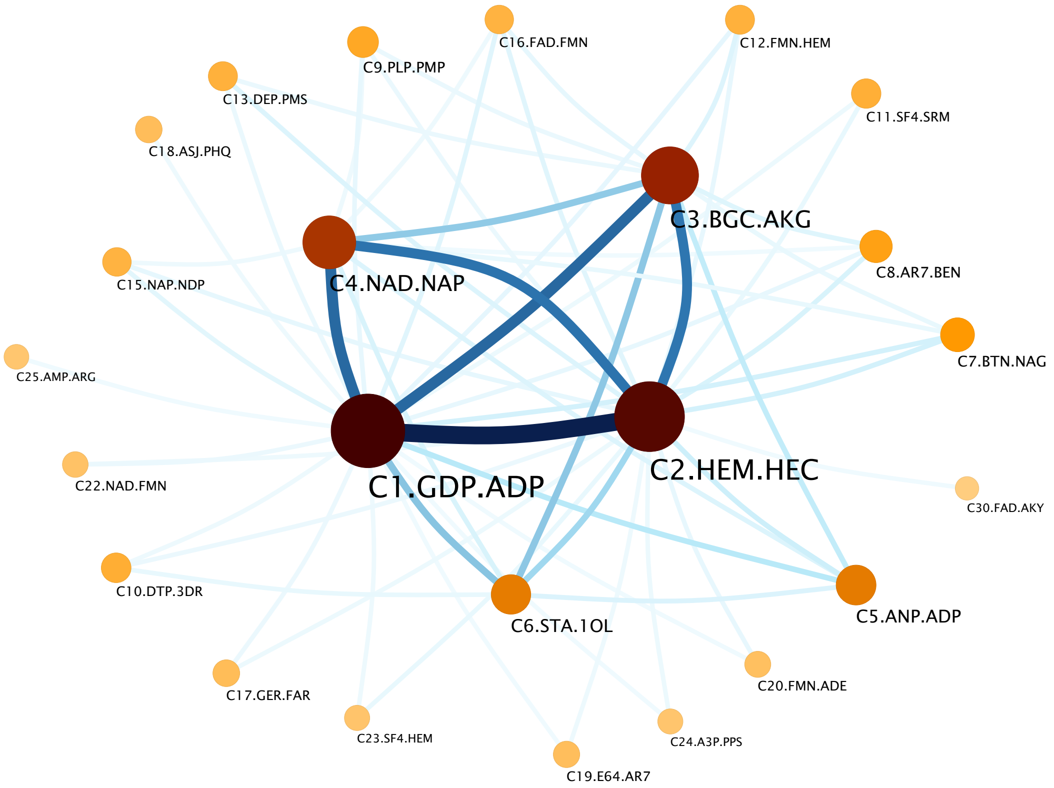

Figure S4 Binding site communities obtained with ProBiS and a z-value threshold of 2.0

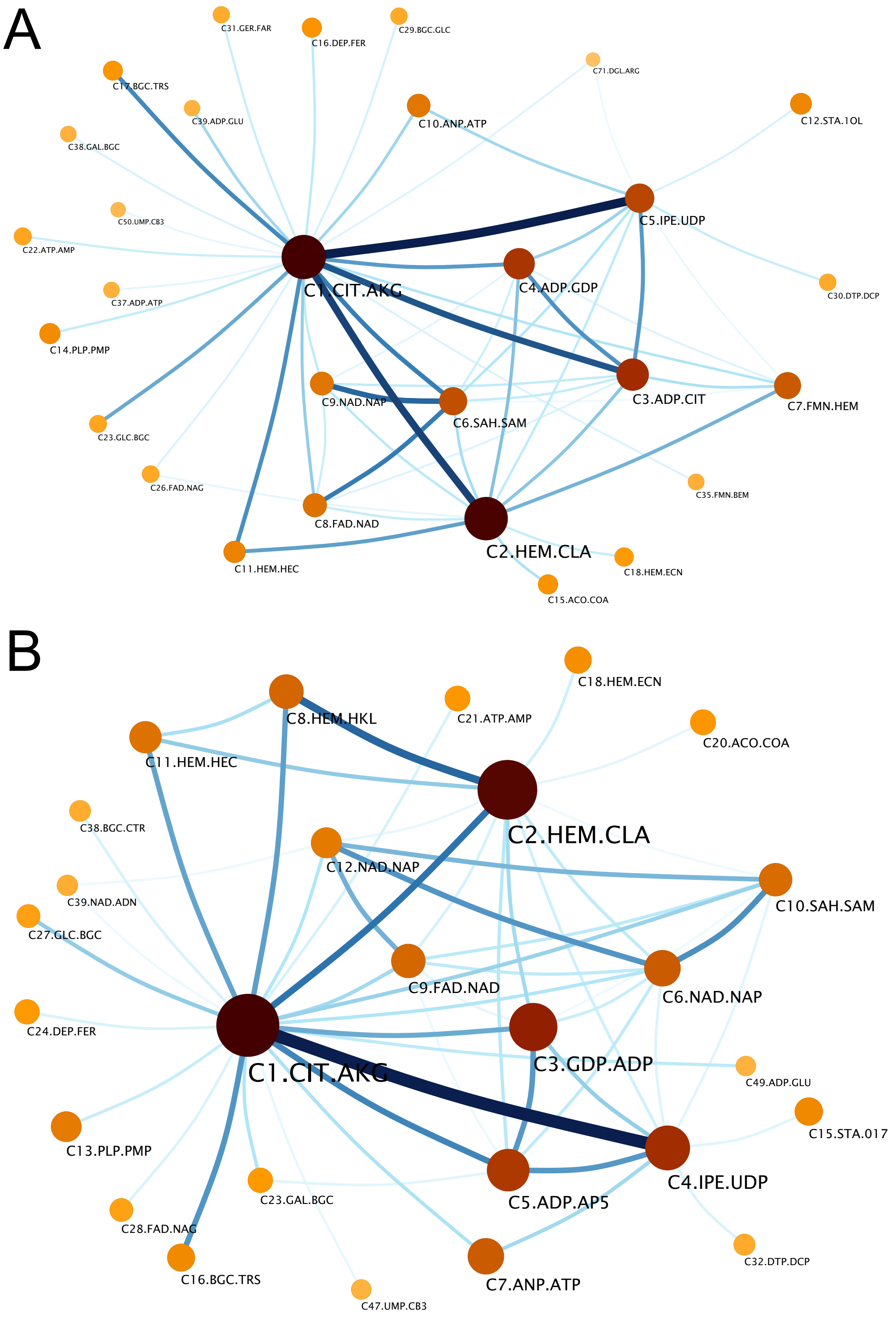

Figure S5 Communities binding site community networks generated with different sequence

identity thresholds. The networks were obtained with sequence identity thresholds of (A) 40% and (B) 90%. We found that, in both cases, the size distributions also follow power-law distributions. The power-law distribution parameters (α, k_{min}) obtained with sequence identities of 40% and 90%, (2.5, 10) and (2.3, 16), are similar to the parameters of the 70% result (2.40, 15). In addition, the overall community structures of the 40% and 90% sequence identity binding site networks are also very similar. The most populated ligands of the largest communities are also conserved or replaced with almost identical ligands. These results indicate that the power-law distribution of community sizes is robust.
